# In Vitro Synergism of Silver Nanoparticles with Antibiotics as an Alternative Treatment in Multiresistant Uropathogens

**DOI:** 10.3390/antibiotics7020050

**Published:** 2018-06-19

**Authors:** Montserrat Lopez-Carrizales, Karla Itzel Velasco, Claudia Castillo, Andrés Flores, Martín Magaña, Gabriel Alejandro Martinez-Castanon, Fidel Martinez-Gutierrez

**Affiliations:** 1 Laboratorio de Microbiología, Universidad Autónoma de San Luis Potosí, San Luis Potosí, CP 78210, Mexico; montsecarrizales@icloud.com (M.L.-C.); kivg56@hotmail.com (K.I.V.); 2 Laboratorio de Células Neurales Troncales, CIACYT-Facultad de Medicina, Universidad Autónoma de San Luis Potosí, San Luis Potosí, CP 78210, Mexico; claudiacastillo@gmail.com; 3 Hospital Central Dr. Ignacio Morones Prieto, San Luis Potosí, CP 78290, Mexico; santosf2000@yahoo.com (A.F.); mmaganaa@hotmail.com (M.M.); 4 Facultad de Estomatología, Universidad Autónoma de San Luis Potosí, San Luis Potosí, CP 78290, Mexico; mtzcastanon@fciencias.uaslp.mx

**Keywords:** antimicrobial activity, biofilm, urinary infection, silver nanoparticles, bacterial resistance

## Abstract

The increase in the prevalence of bacterial resistance to antibiotics has become one of the main health problems worldwide, thus threatening the era of antibiotics most frequently used in the treatment of infections. The need to develop new therapeutic strategies against multidrug resistant microorganisms, such as the combination of selected antimicrobials, can be considered as a suitable alternative. The in vitro activities of two groups of conventional antimicrobial agents alone and in combination with silver nanoparticles (AgNPs) were investigated against a set of ten multidrug resistant clinical isolate and two references strains by MIC assays and checkerboard testing, as well as their cytotoxicity, which was evaluated on human fibroblasts by MTT assay at the same concentration of the antimicrobial agents alone and in combination. Interesting results were achieved when the AgNPs and their combinations were characterized by Dynamic Light Scattering (DLS), Zeta Potential, Transmission Electron Microscopy (TEM), UV–visible spectroscopy and Fourier Transforms Infrared (FTIR) spectroscopy. The in vitro activities of ampicillin, in combination with AgNPs, against the 12 microorganisms showed one Synergy, seven Partial Synergy and four Additive effects, while the results with amikacin and AgNPs showed three Synergy, eight Partial Synergy and one Additive effects. The cytotoxic effect at these concentrations presented a statistically significant decrease of their cytotoxicity (*p* < 0.05). These results indicate that infections caused by multidrug resistant microorganisms could be treated using a synergistic combination of antimicrobial drugs and AgNPs. Further studies are necessary to evaluate the specific mechanisms of action, which could help predict undesirable off-target interactions, suggest ways of regulating a drug’s activity, and identify novel therapeutic agents in this health problem.

## 1. Introduction

Urinary tract infections (UTIs) are defined as inflammatory processes related to the invasion and multiplication of microorganisms that occur at any level of the urinary tract, including urethral (urethritis), bladder (cystitis), ureters (ureteritis) and kidney infections (pyelonephritis) [1].

Approximately between 150 and 250 million cases of UTIs occur each year worldwide [2]. In 2011, more than 8 million cases were reported in the U.S. [1], of which 93,300 were acquired in intensive care units [3]. Recently, the epidemiological bulletin of the Ministry of Health reported in 2016 a total of 4,023,432 cases of UTIs in Mexico, of which 76.68% were in women and only 23.31% in men.

Gram-negative intestinal bacteria are the most common etiology agents of UTIs, where uropathogenic *Escherichia coli* (UPEC) is the major microorganism isolate, which is a member of the Enterobacteriacea family [4]. Other commonly associated pathogens include *Klebsiella* sp. and *Proteus mirabilis*, both of which are characterized by their urease enzyme and Gram-positive bacteria such as *Staphylococcus saprophyticus* and *Enterococcus faecalis* [5].

Urinary Tract Infections Associated with Catheters (CAUTI) are one of the most frequent explanations of nosocomial infections [6]. Patients with urinary catheters show an increment of bacteriuria in relation to duration of catheterization [7], however, the most important factor is the biofilm formation along the catheter surface [7,8]. A biofilm is a resistance mechanism that consists in a self-organized community of microorganisms embedded in a matrix of extracellular polymeric substances synthesized by themselves [9]. Many bacterial species show growth in the form of biofilms, which gives them various advantages [10]. Some of the benefits are metabolic cooperation (nutrients) [11], horizontal gene transfer [12], protection against environmental stresses, lower susceptibility to antimicrobial agents [13,14] and prevention of host defense mechanisms (immune system) [15]. The most common organisms that contaminate the urinary catheter and develop biofilms are strains of *Escherichia coli*, *Pseudomonas aeruginosa*, *Enterococcus*, *Proteus mirabilis*, *Klebsiella pneumoniae* and coagulase-negative staphylococci [10,16].

The antibiotic resistance of bacteria is a global health problem that is continually expanding, and is recognized as a medical problem that increases morbidity and mortality rates, which implies length of hospital stays as well as cost and bad prognosis [17,18]. In fact, the speed at which bacteria are establishing resistance to current antibiotics is faster than the development of new molecules with antimicrobial features. Unfortunately, it is very difficult to identify new bacterial targets that can be used to develop new classes of antimicrobial agents that are safe and effective.

In this context, nanotechnology opens new possibilities, allowing new solutions with old resources. Nanoscale materials such as silver nanoparticles (AgNPs) have emerged as novel agents due to their unique physicochemical properties and remarkable antimicrobial activities that confer a great advantage for the development of alternative products against, for example, multi-drug resistant microorganisms [19,20]. Due to the above, it has been proposed to implement the use of AgNPs on different devices for medical use. One of the strategies is the modification of surfaces of the devices to inhibit the formation of bacterial biofilms [21].

Recently, several studies have indicated that AgNPs can enhance the effect of antibiotics against susceptible and resistant bacteria, [22] as a well as decrease bacterial adhesion in the early stages of biofilm formation. In 2016, Rajendran and et al., impregnated urinary catheters with antibiotics (amikacin and nitrofurantoin) and a synergistic combination of antibiotics and AgNPs (synthesized by a biological method) in order to evaluate antibiofilm activity. The authors reported that the synergistic combination showed a 90% inhibition of bacterial adhesion, whereas functionalization with antibiotics showed only 25% inhibition [23].

Some authors have reported that AgNPs have toxic effects on mammalian cells; for example, impairment of normal mitochondrial function, increased membrane permeability and generation of reactive oxygen species [24,25].

In this study, the synergistic activity of AgNPs was evaluated with conventional antibiotics against Gram-positive and Gram-negative multi-drug resistant isolates from clinical samples. Results presented here show that AgNPs, in combination with antibiotics, increase the antimicrobial effect in an additive or synergistic manner. Furthermore, MTT assays suggest that at low concentrations the AgNPs and their combinations do not present cytotoxic effects in eukaryotic cells. 

## 2. Results and Discussion

### 2.1. Synthesis and Characterization of AgNPs

**Figure 1:**
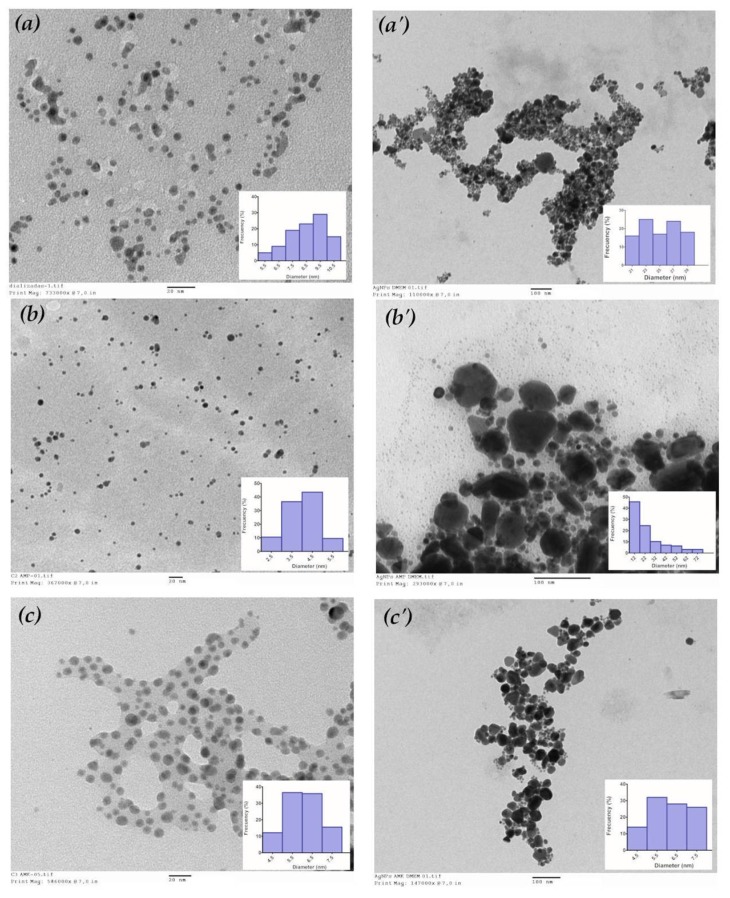
Morphological characterization of silver nanoparticles and their combinations with antibiotics. Transmission electron micrographs showing the formation of spherical and pseudospherical nanoparticles. (**a**) AgNPs; (**a’**) AgNPs in DMEM; (**b**) AgNPs + AMP; (**b’**) AgNPs + AMP in DMEM; (**c**) AgNPs + AMK; (**c’**) AgNPs + AMK in DMEM. Insets: Particle size distribution histogram. DMEM: Dulbecco’s Modified Eagle Medium. AMP: Ampicillin. AMK: Amikacin.

TEM micrograph revealed that the AgNPs were of spherical and pseudospherical shapes (Figure 1). Based on the particle size distribution histogram evaluated from the corresponding TEM micrograph (n = 100), the mean (±SD) size of AgNPs 8.57 ± 1.17 nm was calculated. The mean size for the combination of AgNPs with ampicillin (AgNPs + AMP) was 4.01 ± 0.80 nm and for the combination of AgNPs with amikacin (AgNPs + AMK) was 6.03 ± 0.87 nm (Table 1).

The particle size distributions of AgNPs + AMP and AgNPs + AMK were also evaluated in Dulbecco’s Modified Eagle Medium (DMEM).

AgNPs synthesized in aqueous solution and their combinations with antibiotics were characterized by DLS (Table 1). The results of the dialyzed AgNPs showed a narrow size distribution with a hydrodynamic diameter of (±SD) 8.23 nm ± 0.91 nm and a zeta potential value of −40.80 mV ± 9.54 mV. The combination of AgNPs + AMP also showed a narrow size distribution with a hydrodynamic diameter of 4.69 nm ± 0.51 nm. This decrease in size was attributed to the fact that ampicillin favors the homogeneous dispersion of the nanoparticles and gives it a greater stability when it obtains a zeta potential value of −51.00 mV ± 20.20 mV. The results of combination of AgNPs + AMK showed a narrow distribution of sizes with a hydrodynamic diameter of 947.90 nm ± 65.30 nm and a value of −21.10 mV ± 4.63 mV for zeta potential. The increase of size of the nanoparticles are attributed to the addition of amikacin, which favors agglomeration of the AgNPs and causes an increase in the zeta potential, which translates in less stable nanoparticles.

The hydrodynamic diameters and zeta potentials of AgNPs and their combinations were also evaluated in DMEM.

**Figure 2:**
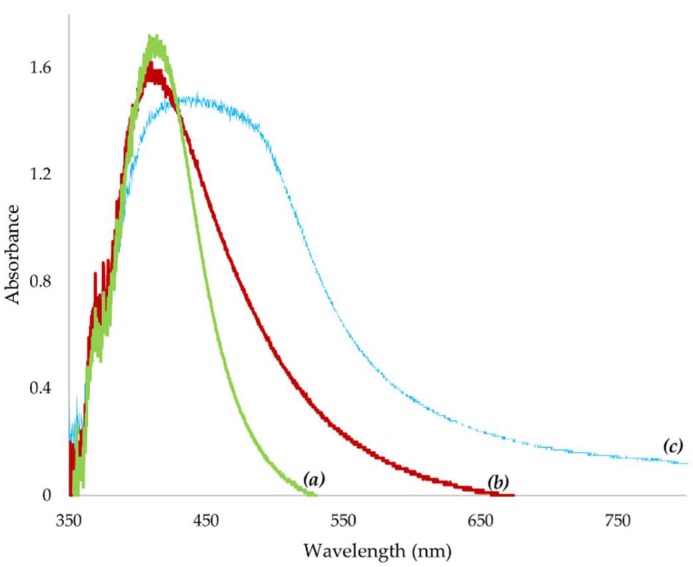
UV–visible absorption spectra of silver nanoparticles and their combinations with antibiotics. UV–visible spectrum showed the maximum absorbance at (**a**) 412 nm for AgNPs, (**b**) 410 nm for AgNPs + AMP and (**c**) 450 nm for AgNPs + AMK. AMP: Ampicillin. AMK: Amikacin.

The UV–visible spectrum revealed a peak at a wavelength of 412 nm for AgNPs whereas in the combination AgNPs + AMP, the peak was observed at 410 nm and in the combination AgNPs + AMK, the peak appeared at a wavelength of 450 nm (Figure 2). These peaks correspond to the excitation of the surface plasmon of AgNPs. The plasmonic resonance depends on several parameters, like the nature, size and geometry of the nanoparticles and the physical properties of the medium in which the nanoparticles are dispersed. In the case of AgNPs, the plasmon peak appears at a wavelength around 400 nm [26].

In this study, gallic acid was used as a reducing and stabilizing agent, a molecule with a carboxylic group and rich in hydroxyl functional groups. Yoosaf et al. proposed that AgNPs are stabilized by gallic acid through electrostatic interactions through their oxidized carboxylic group and the afore-cited hydroxyl groups were capable of forming hydrogen bonds [27]. 

**Figure 3:**
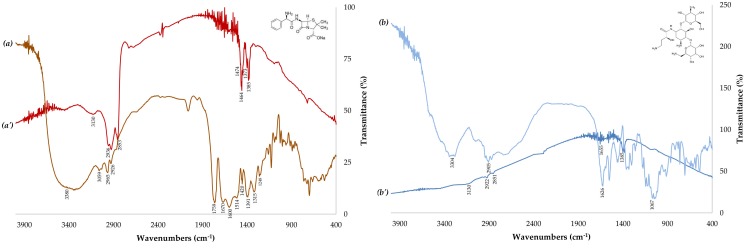
FTIR spectra of the antibiotics and their combinations with silver nanoparticles. (**a**) AMP: Ampicillin; (**a’**) AgNPs + AMP; (**b**) AMK: Amikacin; (**b’**) AgNPs + AMK. Insets: AMP and AMK structure.

To study the possible interactions between antibiotics and the surface of the AgNPs, FTIR was performed (Figure 3). FTIR is useful for determining the chemical composition of antibiotics involved in the coating of AgNPs. The observed intense bands were compared with standard values to identify the functional groups. The FTIR spectra of the antibiotics (Figure 3a,b) changed greatly upon the combination with AgNPs (AgNPs + AMP and AgNPs + AMK), as displayed in Figure 3a’,b’. Ampicillin is a molecule that has carbonyl and amine functional groups, while amikacin is a molecule rich in hydroxyl and amine groups. In the case of ampicillin, the band at 1759 cm^−1^ disappeared completely (Figure 3a’), which suggests that the antibiotic interacts with the AgNPs through its carbonyl group (C=O). Furthermore, the peak of the primary amine at 3380 cm^−1^ (Figure 3a) shifted to 3130 cm^−1^ (Figure 3a’) after the combination with nanoparticles, indicating that the amine functional group was involved in the interaction with the surface of the AgNPs. Therefore, the spectrum of the amikacin showed that the bands at 1626 cm^−1^ corresponding to a carbonyl group and 3400 cm^−1^ for a primary amine disappeared completely (Figure 3b’) after being combined with the nanoparticles. Those results of the FTIR suggest that the functional groups of the antibiotics could be involved in the interaction by hydrogen bonds with gallic acid [28,29]. The results showed in Figure 3 are in concordance with the previous results of the Hua Deng et al., 2016, who carried out UV–Vis and Raman spectroscopy studies reveal that amikacin can form complexes with AgNPs, while ampicillin do not [30]. The authors reported that no Raman enhancement is observed when AgNPs are combined with ampicillin at any test concentrations. This implies that the antibiotics do not strongly interact with AgNPs to replace the stabilizer molecules on the surface of AgNPs. Moreover, they infer that the combinations of antibiotics and AgNPs have different ways to develop antimicrobial activities.

### 2.2. Samples and Bacterial Strains 

The multidrug resistance clinical strains used for this experiment were isolated from the urine of patients with CAUTI; microbiological analysis showed that the clinical pathogenic strains isolated were in accordance with the main etiologic agents causing CAUTI; these results are in accordance with previously results reported when the UTI were evaluated on hospitalized patients in Kolkata, an eastern region of India, as well as in studies where complicate and non-complicate UTI were studied [4,31].

### 2.3. Antimicrobial Test

A set of ten clinical pathogenic strains resistant to antibiotics associated with CAUTI were evaluated, of which two corresponded to Gram-positive strains and the rest to Gram-negative strains. The results showed that all clinical isolates (*E. faecium*, *S. aureus*, *A. baumannii*, *E. cloacae*, three different isolates of *E. coli*, *K. pneumoniae*, *M. morgannii* and *P. aeruginosa*) showed a MIC to AgNPs between 4 and 16 μg/mL. The bacterial strains showed MIC values of 4–128 μg/mL for amikacin and all Gram-negative strains were resistant to ampicillin. 

### 2.4. Checkerboard Synergy

Table 2 shows the MIC archived with the ten multidrug resistance clinical strains grown in Mueller Hinton Broth with amikacin and ampicillin, both in the absence of AgNPs and when present; when the bacteria were incubated with the combination of AgNPs and antibiotic (AgNPs + AMK and AgNPs + AMP), the ampicillin and amikacin MICs decreased drastically for all strains. The combination of AgNPs + AMK reduce the MIC by 2 to 32-fold. By contrast, with the combination of AgNPs + AMP reduce the MIC just with *S. aureus* and *E. cloacae* by 1- and 4-fold respectively; with the other microorganisms, the MICs were reduced by 16- and 32-fold. These results show a better antimicrobial activity of the combination of AgNPs + AMP, which could be explained by the interaction between the AgNPs and ampicillin and, therefore, arrangement of the molecules in a new compound, which could work in both ways, like independent chemical entities or a new compound; more experiments are needed to explain the role and proportion of each one of them.

**Figure 4:**
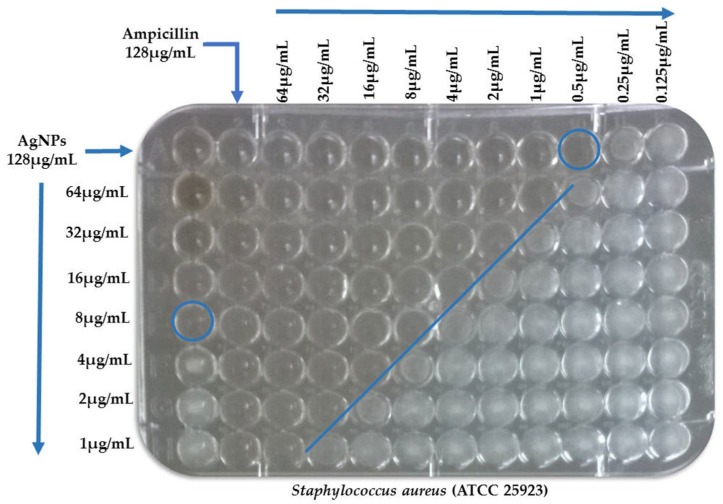
Example of checkerboard testing. Blue circles denote the MIC of antimicrobial agents (alone) and blue line denote the FIC (combination of both). MIC: Minimum Inhibitory Concentration. FIC: Fractional Inhibitory Concentration.

The synergistic effects of AgNPs and conventional antibiotics were evaluated by determination of the Fractional Inhibitory Concentration (FIC) index (Figure 4). Synergistic interactions of AgNPs and amikacin were observed against *Acinetobacter baumannii*, *Escherichia coli* (508) and *E. coli* (ATCC 25922). Synergistic interactions of AgNPs and ampicillin were found only against *Acinetobacter baumannii*. Other combinational activities of AgNPs and antibiotics were considered as partially synergistic interactions. These synergistic activities of AgNPs in combination with conventional antibiotics suggest that it may be possible to reduce the viability of bacterial strains at lower antibiotic concentrations (Table 3).

### 2.5. Cytotoxicity of AgNPs

**Figure 5:**
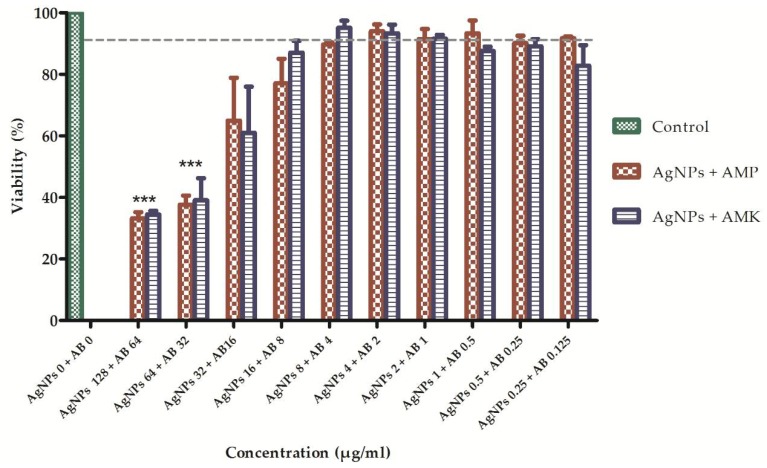
Viability of cells treated with combinations of silver nanoparticles and antibiotics. To measure cytotoxicity, fibroblasts were treated with increasing concentrations of AgNPs + AMP (red) or AgNPs + AMK (blue) (n = 3). Twenty-four hours after of addition of treatment cell viability was determined using MTT. Results are expressed as mean and standard deviation. * *p* < 0.05, ** *p* < 0.01, *** *p* < 0.001 by two-way ANOVA. Control: DMEM. AB: Antibiotic = Amikacin (AMK) or Ampicillin (AMP).

The cytotoxicity of AgNPs and antibiotics was evaluated separately and in combination by the MTT assay. AgNPs were tested at concentrations of 0.25, 1, 4, 16, 64 and 128 μg/mL in human fibroblasts. The percentages of living and dead cells were determined after 24 h of being exposed in contact with the AgNPs. AgNPs concentrations less than 4 μg/mL showed a cytotoxic effect that resulted in a death rate of 13.8% or less. However, concentrations greater than 64 μg/mL caused significant cell death of approximately 67%. In addition, antibiotics (ampicillin and amikacin) were tested at concentrations of 64, 32, 8, 2, 0.5 and 0.125 μg/mL in human fibroblasts. It was found that the viability percentage for each of the concentrations of ampicillin was greater than 80% and for amikacin greater than 76%. To evaluate the cytotoxic effects of the combination of AgNPs and conventional antibiotics, ten combinations of different concentrations (AgNPs μg/mL + antibiotic μg/mL: 128 + 64; 64 + 32; 32 + 16; etc.) were tested. It was found that there is no statistically significant difference between the two treatments (AgNPs + AMP and AgNPs + AMK) with respect to cell viability when the two-way ANOVA was performed (*p* < 0.05). However, combinations of 128 μgAgNPs/mL + 64 μg of antibiotic/mL and 64 μgAgNPs/mL + 32 μg of antibiotic/mL caused a statistically significant decrease in cell viability when compared with the rest of the combinations tested (*p* < 0.05) evidenced by the reduction of the mitochondrial activity. On the other hand, it is important to highlight that when AgNPs were combined with antibiotics, at concentrations equal to or less than 8 μg AgNPs/mL showed a viability percentage between 90–95% (Figure 5).

Interesting results were archived when two specific multi-resistant strains, *E. faecium* with resistance to vancomycin and *A. baumannii* with resistance to meropenem, were tested and both showed, with the combination of AgNPs + AMK, a reduction of the MIC by 32-fold, as well as with the combination of AgNPs + AMP of the MIC by 16-fold. In both cases, the cytotoxicity to fibroblasts, of the concentrations of AgNPs + Antibiotic which showed a reduction of MIC, showed a reduction with statistical significance (Table 4).

## 3. Materials and Methods 

### 3.1. Synthesis of AgNPs

For the synthesis of nanoparticles, AgNO_3_ (0.01 M) was used as a metallic precursor and gallic acid (0.1 g) was used as a reducer and stabilizer agent. NaOH (3 M) was used for pH regulation. AgNPs were synthesized by dissolving 0.0169 g of AgNO_3_ in 90 mL of deionized water and this solution was placed in a 250 mL reaction vessel. A total of 0.01 g gallic acid was dissolved in 10 mL of deionized water and was added to the AgNO_3_ solution with magnetic stirring. After the addition of gallic acid, the pH value of the solution was adjusted using a solution of NaOH 3 M. At the end of the synthesis, approximately 100 mL of nanoparticles were obtained with a pH of 12.66, of which 50 mL were dialyzed for 48 h on a 12 kDa nitrocellulose membrane.

### 3.2. Characterization of AgNPs

AgNPs were characterized by Dynamic Light Scattering (DLS), the hydrodynamic diameter and zeta potential were determined using a Malvern Zetasizer Nano ZS (Malvern Panalytical, Malvern, United Kingdom) operating with a He-Ne laser at a wavelength of 633 nm and a detection angle of 90°. Samples were analyzed for 60 s at 25 °C. To confirm the shape, each sample was diluted with deionized water and 50 μL of each suspension was placed on a copper wire for Transmission Electron Microscopy (TEM). All samples were analyzed by Transmission Electron Microscopy (JEOL JEM-1230, Tokyo, Japan) at an acceleration voltage of 100 kV. Afterwards, AgNPs were characterized by UV-visible spectroscopy using an S2000 UV-Vis spectrophotometer from OceanOptics Inc. (Dunedin, FL, USA). The functional groups present in the antibiotics were identified by Fourier Transform Infrared Spectroscopy (Shimadzu, IRaffinity-1, Osaka, Japan). A certain amount of nanopowder was collocated in the equipment and the spectrum was taken in the range of 400–4000 cm^−1^ with a resolution of 2 cm^−1^ and 200 times scanning using the attenuated total reflection (ATR) method.

### 3.3. Preparation and Characterization of Combinations of AgNPs with Antibiotics

To study the effect of ampicillin and amikacin on the size, shape and stability of the AgNPs, an aqueous solution containing a 1:1 ratio of antibiotic (128 μg/mL) and nanoparticles (128 μg/mL) was prepared for each antibiotic. These solutions were characterized by TEM, DLS, zeta potential and UV-visible spectroscopy. On the other hand, the chemical interaction between the AgNPs and antibiotics was carried out by FTIR, we prepared an aqueous solution containing higher concentrations of antimicrobials (500 μg/mL), the combinations preserved the ratio of 1:1. Subsequently, these solutions were centrifuged, keeping only the precipitate, which was left to dry for 24 h at room temperature.

### 3.4. Patients

The study protocol and the letter of informed consent were approved by the local Hospital’s Ethics and Science Committee with the number 102-16.

The study included urine samples from patients with urinary catheters treated at the local Hospital. Patients were selected in basis to NOM-045-SSA2-2005. For the purposes of this study, only samples of patients older than 15 years, of any genus, whose culture had a bacterial count greater than or equal to 50,000 CFU/mL were selected, and also with an antimicrobial resistance profile.

### 3.5. Microbiological Analysis

#### 3.5.1. Sample Collection and Bacterial Culture

Urine samples were collected in 5 mL syringes using the probe puncture technique under aseptic conditions by trained personnel. Microbiological analysis was performed according to the established criteria by the American Society for Microbiology (ASM) for urine samples. A count greater than or equal to 50 colonies (equivalent to 50,000 CFU/mL) was considered as the cutoff point for diagnosing infection. Plaques without development at 24-h incubation were discarded and indicated absence of urinary tract infection.

#### 3.5.2. Identification and Antimicrobial Susceptibility Profile of Clinical Strains

The identification and antimicrobial susceptibility of the microorganisms isolated were determined by VITEK2 equipment. From a pure culture grown for 24 h, an inoculum was transferred to a test tube with 3 mL of solution sterile saline (0.45% to 0.5% NaCl aqueous solution, pH 4.5 to 7.0). Subsequently, the turbidity was adjusted to 0.50–0.63 units of the McFarland scale with the densitometer. The bacterial suspension was placed inside the cassette. The identification and susceptibility cards were placed in the nearby slot, inserting the transfer tube into the test tube with the corresponding suspension. The cassette was placed with the samples in the VITEK2 system. The resistance profiles are shown in Table S1. 

#### 3.5.3. Conservation of Strains

The conservation of the strains was carried out by the freeze conservation method. Glycerol was used as the cryoprotective agent. The samples were stored at −20 °C.

### 3.6. Antimicrobial Test

The Minimum Inhibitory Concentration (MIC) was determined by broth microdilution assay in accordance with the procedures recommended by the Clinical and Laboratory Standards Institute (CLSI) [34]. MICs were determined by incubating the microorganisms in 96-well microplates for 24 h at 37 °C. Microorganisms were exposed to serial dilutions of the antimicrobial agent (AgNPs, ampicillin and amikacin), and the end points were determined when no turbidity in the well was observed. The standardization of the method was made based on the criteria established by the CLSI. The strains used were *Staphylococcus aureus* ATCC 25923 and *Escherichia coli* ATCC 25922. The antibiotics used were oxacillin (64 μg/mL) and ceftazidime (32 μg/mL). The assay was performed in duplicate for four days. The results of the standardization of the MIC are shown in Table S2. 

### 3.7. Checkerboard Synergy

The MIC of each antimicrobial substance alone or in combination was determined by a broth microdilution method in accordance with CLSI standards. The assay was performed in 96-well microtiter plates, a two-fold dilution of the antibiotic was distributed into each well to obtain a varying concentration of 128, 64, 32, 16, 8, 4, 2, 1, 05, 0.25 and 0.125 μg/mL in the wells of the first row, while those of the AgNPs were similarly distributed among the first column (128 to 1 μg/mL). The AgNPs dilutions were started from the columns to the right and the antibiotic dilutions were started from the first row downwards. Thus, each of the wells held a unique combination of concentrations of AgNPs and the antibiotic. The broth microdilution plates were inoculated with each test microorganism to yield the appropriate density (10^5^ CFU/mL) in 100 μL Mueller–Hinton broth and incubated at the optimum temperature and time of growth conditions (37 °C/24 h). The MIC was determined as the least dilution without any turbidity. The MICs of single antimicrobial A and B (MIC_A_ and MIC_B_) and in combination were determined. The ten multidrug resistant clinical strains were exposed to serial dilutions of the antimicrobial agents. Subsequently, we calculated the proportion: MIC_Antibiotic alone_/MIC_Antibiotic in combination_ (fold-change) to describe the number of times that MIC decreased from an initial to final value.

### 3.8. Cytotoxicity of AgNPs

To evaluate the toxicity of AgNPs in combination with antibiotics, human fibroblasts (baby foreskin) were used. Cells were dispensed in 96-well microplates at a density of 5000 cells per well in DMEM supplemented with 1% fetal bovine serum (FBS) for 24 h at 37 °C. After 24 h the cells were incubated with the established concentrations of AgNPs and antibiotics for 24 h at 37 °C. Treatment was withdrawn and 100 μL MTT (3-(4,5-cimethylthiazol-2-yl)-2,5-diphenyl tetrazolium bromide) (0.5 mg/mL) was added in DMEM without FBS for 4 h at 37 °C for the formation of formazan crystals. MTT was removed from the wells and 100 μL of DMSO was added to read absorbance in a Synergy H1 microplate reader with Gen 5 software (Biotek Instruments, Winooski, VT, USA) at a wavelength of 595 nm. As a positive control, cells were treated with hydrogen peroxide and as a negative control, they were only treated with medium. The assay was performed in triplicate. 

### 3.9. Statistical Analysis

Each assay was repeated three times. Data are presented as mean ± standard deviation (SD). The comparison between the effects of the two sources of variation was made using the two-way analysis of variance (ANOVA). The analysis was performed with the statistical software SPSS 23.0 (IBM, New York, NY, USA). A value of *p* < 0.05 was considered significant. 

## 4. Conclusions

These results indicate that infections due to multidrug resistant microorganisms could be treated by the use of a synergistic combination of antimicrobial drugs and AgNPs. Further studies are necessary to evaluate the specific mechanisms of action, which could help predict undesirable off-target interactions, suggest ways of regulating a drug’s activity, and identify novel therapeutics to this health problem.

## Figures and Tables

**Table 1:** Characterization of silver nanoparticles and their combinations with antibiotics by DLS and TEM.

	DLS				TEM	
	Hydrodynamic Diameter (nm)	Hydrodynamic Diameter in DMEM (nm)	Zeta Potential (mV)	Zeta Potential in DMEM (mV)	Diameter (nm)	Diameter in DMEM (nm)
AgNPs	8.23 ± 0.91	39.25 ± 6.42	−40.80 ± 9.54	−16.20 ± 0.0	8.57 ± 1.17	25.08 ± 2.74
AgNPs + AMP	4.69 ± 0.51	26.25 ± 5.55	−51.00 ± 20.20	−15.60 ± 0.0	4.01 ± 0.80	24.17 ± 16.29
AgNPs + AMK	947.90 ± 65.30	222.50 ± 47.10	−21.10 ± 4.63	−18.10 ± 7.56	6.03 ± 0.87	6.14 ± 0.99

Data are expressed as mean and standard deviation. DLS: Dynamic Light Scattering. TEM: Transmission Electron Microscopy. DMEM: Dulbecco’s Modified Eagle Medium. AMP: Ampicillin. AMK: Amikacin.

**Table 2:** Efficacy of silver nanoparticles, antibiotics and their combinations against clinical strains.

Clinical Strains	MIC (µg/mL)			Fold Change	MIC (µg/mL)		Fold Change
	AgNPs	AMK	AgNPs + AMK *		AMP	AgNPs + AMP *	
**Gram-positive**							
*Enterococcus faecium*	8	128	4	32	128	8	16
*Staphylococcus aureus*	8	4	2	2	4	4	1
**Gram-negative**							
*Acinetobacter baumannii*	16	128	4	32	128	8	16
*Enterobacter cloacae*	16	16	8	2	128	32	4
*Escherichia coli* (501) **	8	64	4	16	128	8	16
*Escherichia coli* (508) **	8	4	1	4	128	8	16
*Escherichia coli* (515) **	8	32	8	4	128	8	16
*Klebsiella pneumoniae*	4	4	2	2	128	4	32
*Morganella morganii*	8	8	4	2	128	8	16
*Pseudomonas aeruginosa*	4	32	4	8	128	4	32

* Minimum Inhibitory Concentration (MIC) represents the concentration of antibiotic (amikacin or ampicillin) present in the combination. AMK: Amikacin. AMP: Ampicillin. ** The numbers in parentheses indicate that *E. coli* corresponds to a different clinical sample.

**Table 3:** FIC index of combinations among silver nanoparticles and antibiotics against clinical and reference strains.

Bacterial Strains	FIC Index			
	AgNPs + AMK		AgNPs + AMP	
**Gram-positive**				
*Staphylococcus aureus* (ATCC 25923)	1.06	(AD)	1.03	(AD)
*Enterococcus faecium*	0.53	(PS)	0.56	(PS)
*Staphylococcus aureus*	0.63	(PS)	1.25	(AD)
**Gram-negative**				
*Escherichia coli* (ATCC 25922)	0.31	(S)	1.50	(AD)
*Acinetobacter baumannii*	0.28	(S)	0.31	(S)
*Enterobacter cloacae*	0.75	(PS)	1.25	(AD)
*Escherichia coli* (501) **	0.56	(PS)	0.56	(PS)
*Escherichia coli* (508) **	0.31	(S)	0.56	(PS)
*Escherichia coli* (515) **	0.75	(PS)	0.56	(PS)
*Klebsiella pneumoniae*	0.75	(PS)	0.53	(PS)
*Morganella morganii*	0.75	(PS)	0.56	(PS)
*Pseudomonas aeruginosa*	0.63	(PS)	0.53	(PS)

FIC: Fractional Inhibitory Concentration. AMK: Amikacin. AMP: Ampicillin. ATCC: American Type Culture Collection. The FIC index was interpreted as follows: FIC ≤ 0.5, Synergy (S); 0.5 ≤ FIC < 1, Partial Synergy (PS); FIC = 1, Additive (AD); 2 ≤ FIC < 4, Indifferent (I); FIC > 4, Antagonism (AN) [32,33]. ** The numbers in parentheses indicate that *E. coli* corresponds to a different clinical sample.

**Table 4:** Viability of fibroblasts treated with silver nanoparticles, antibiotics and their combinations using concentrations corresponding to the MIC value.

Clinical Strains	Viability of Fibroblasts (%)				
	AgNPs	AMK	AgNPs + AMK	AMP	AgNPs + AMP
*E. faecium*	>80	≈55	>90	>90	85–95
*A. baumannii*	72	≈55	>90	>90	85–95

MIC: Minimum Inhibitory Concentration. AMK: Amikacin. AMP: Ampicillin.

## Supplementary Materials

The following are available online at http://www.mdpi.com/2079-6382/7/2/50/s1, Table S1: Resistance profile of the set of clinical strains, Table S2: Standardization of the method MIC.

## Appendix A. FIC Calculation

One strategy used to overcome the resistance mechanism of the microorganisms is the use of the combination of drugs. The way to measure their effectiveness is through the calculation of the Fractional Inhibitory Concentration (FIC) index.

The FIC index was calculated as follows:

FIC of AgNPs = MIC_AgNPs_ in combination/MIC_AgNPs_ alone
FIC of Antibiotic = MIC_Antibiotic_ in combination/MIC_Antibiotic_ alone
FIC index = FIC of AgNPs + FIC of Antibiotic

The FIC index was interpreted as follows: FIC < 0.5, Synergy (S); 0.5 ≤ FIC <1, Partial Synergy (PS); FIC = 1, Additive (AD); 2 ≤ FIC < 4, Indifferent (I); FIC > 4, Antagonism (AN).
