# Supplementary material for: In Vitro Synergism of Silver Nanoparticles with Antibiotics as an Alternative Treatment in Multiresistant Uropathogens
Source: Antibiotics (Basel). 2018 Jun 19;7(2):50. doi: 10.3390/antibiotics7020050 (PMC6023009; doi:10.3390/antibiotics7020050)
Supplement: Supplementary file 1 [file antibiotics-07-00050-s001.zip › Table -s/Table S1_Resistance profile of the set of clinical strains.docx]

**Table S1.** Resistance profile of the set of clinical strains – Gram-negative.

| Antibiotics | Gram-negative | | | | | | | |
| --- | --- | --- | --- | --- | --- | --- | --- | --- |
|  | ***K. pneumoniae*** | ***E. cloacae*** | ***E. coli* (501)** | ***M. morganii*** | ***P. aeruginosa*** | ***E. coli* (508)** | ***A. baumannii*** | ***E. coli* (515)** |
| Amikacin | S | S | S | S | R | S | R | S |
| Ampicillin | R | R | R | R |  | R |  | R |
| Ampicillin/Sulbactam | R | R | R | R |  | R | R | R |
| Aztreonam | R | R | R |  | R | R | R | R |
| Cefazolin | R | R | R | R |  | R |  | R |
| Cefepime | R | S | R | I | R | R | R | R |
| Ceftriaxone | R | R | R | S |  | R | R | R |
| Ciprofloxacin | I | S | R | S | R | R | R | R |
| Ertapenem | S | S | S | S |  | S |  | S |
| Gentamicin | R | S | R | S | R | R | R | R |
| Meropenem | S | S | S | S | R | S | R | S |
| Nitrofurantoin | I | S | S | R | R | R | R | I |
| Piperacillin/Tazobactam | S | R | R |  |  | S |  | S |
| Tigecycline | S | S | S | R |  | S | S | S |
| Trimethoprim/Sulfamethoxazole | R | S | R |  |  | R | R | R |

S: Sensitive. I: Intermediate. R: Resistant.

**Table S1.** Resistance profile of the set of clinical strains – Gram-positive.

| Antibiotics | Gram-positive | |
| --- | --- | --- |
|  | ***E. faecium*** | ***S. aureus*** |
| Ampicillin | R |  |
| Bencylpenicillin | R | R |
| Ciprofloxacin | R | S |
| Clindamycin | R | R |
| Erythromycin | R | R |
| Gentamicin |  | S |
| Levofloxacin | R | S |
| Linezolid | S | S |
| Moxifloxacin | R | S |
| Oxacillin |  | S |
| Quinupristin/Dalfopristin | S | S |
| Rifampicin |  | S |
| Tetracycline | R | S |
| Tigecycline | S |  |
| Trimethoprim/Sulfamethoxazole |  | S |
| Vancomycin | R | S |

S: Sensitive. I: Intermediate. R: Resistant.
