# Supplementary material for: In Vitro Synergism of Silver Nanoparticles with Antibiotics as an Alternative Treatment in Multiresistant Uropathogens
Source: Antibiotics (Basel). 2018 Jun 19;7(2):50. doi: 10.3390/antibiotics7020050 (PMC6023009; doi:10.3390/antibiotics7020050)
Supplement: Supplementary file 1 [file antibiotics-07-00050-s001.zip › Table -s/Table S2_Standardization of the method MIC.docx]

**Table S2.** Standardization of the method MIC.

| Species | Theoric value  CLSI (µg/mL) | | Experimental value^a^  (µg/mL) | | AgNPs^b^  (µg/mL) |
| --- | --- | --- | --- | --- | --- |
|  | **AMK** | **AMP** | **AMK** | **AMP** |  |
| *Staphylococcus aureus* (ATCC 25923) | 1.0 – 4.0 | 0.5 – 2.0 | 1 ± 0 | 0.5 ± 0 | 8 ± 0 |
| *Escherichia coli* (ATCC 25922) | 0.5 – 4.0 | 2.0 – 8.0 | 2 ± 0 | 4 ± 0.94 | 4 ± 0 |

MIC: Minimum Inhibitory Concentration. CLSI: Clinical & Laboratory Standards Institute. ATCC: American Type Culture Collection. AMK: Amikacin. AMP: Ampicillin. ^a, b^ MIC is expressed in average and standard deviation.
